# Predicting parkinsonism side-effects of antipsychotic polypharmacy prescribed in secondary mental healthcare

**DOI:** 10.1177/0269881118796809

**Published:** 2018-09-20

**Authors:** Giouliana Kadra, Athan Spiros, Hitesh Shetty, Ehtesham Iqbal, Richard D Hayes, Robert Stewart, Hugo Geerts

**Affiliations:** 1 King’s College London, Psychological Medicine, Institute of Psychiatry, Psychology and Neuroscience, London, UK; 2 In Silico Biosciences, Berwyn, PA, USA; 3 South London and Maudsley NHS Trust, BRC Nucleus, London, UK; 4 King’s College London, SGDP, Institute of Psychiatry, Psychology and Neuroscience, London, UK

**Keywords:** Antipsychotic polypharmacy, computer-modelling, antipsychotics, concomitant, electronic health records

## Abstract

**Background:**

Computer-modelling approaches have the potential to predict the interactions between different antipsychotics and provide guidance for polypharmacy.

**Aims:**

To evaluate the accuracy of the quantitative systems pharmacology platform to predict parkinsonism side-effects in patients prescribed antipsychotic polypharmacy.

**Methods:**

Using anonymized data from South London and Maudsley NHS Foundation Trust electronic health records we applied quantitative systems pharmacology, a neurophysiology-based computer model of humanized neuronal circuits, to predict the risk for parkinsonism symptoms in patients with schizophrenia prescribed two concomitant antipsychotics. The performance of the quantitative systems pharmacology model was compared with the performance of simple parameters such as: combination of affinity constants (1/K_sum_); sum of D_2_R occupancies (D_2_R) and chlorpromazine equivalent dose.

**Results:**

We identified 832 patients with schizophrenia who were receiving two antipsychotics for six or more months, between 1 January 2007 and 31 December 2014. The area under the receiver operating characteristic (AUROC) curve for the quantitative systems pharmacology model was 0.66 (*p* = 0.01), while AUROCs for D_2_R, 1/K_sum_ and chlorpromazine equivalent dose were 0.52 (*p* = 0.350), 0.53 (*p* = 0.347) and 0.52 (*p* = 0.330) respectively.

**Conclusion:**

Our results indicate that quantitative systems pharmacology has the potential to predict the risk of parkinsonism associated with antipsychotic polypharmacy from minimal source information, and thus might have potential decision-support applicability in clinical settings.

## Introduction

Antipsychotic polypharmacy (APP) use has been discouraged by existing guidelines (Barnes, 2011) due to lack of evidence to support its effectiveness (Kroken et al., 2009; Waddington et al., 1998; Yu et al., 2009) as well as evidence indicating an increased risk for further hospitalizations (Kadra et al., 2017), high dose prescribing (Roh et al., 2013; Torniainen et al., 2015), side-effects (Grundy, 2006) and mortality (Joukamaa et al., 2006; Waddington et al., 1998). However, APP remains prevalent in clinical practice (Gallego et al., 2012; Mace and Taylor, 2015) and therefore there has been an increasing need to better understand the interactions between different antipsychotics.

Previous literature on APP has indicated that side-effects are common (Citrome, 2013) and their nature and severity depend substantially on the dose and nature of the antipsychotics. There are currently no guidelines for APP prescribing with the exception of pharmacokinetic–pharmacokinetic interactions (Kennedy et al., 2013). However, this does not specify which antipsychotics can be combined and at what dose. At present, it is common to grade antipsychotic combinations using chlorpromazine equivalents (Davis, 1976) to estimate the risk for the occurrence of side-effects. While this has been successful for single antipsychotics, the non-linear interaction of two or more antipsychotics at different receptor systems makes this approach less useful for combination treatments.

In this project, we focused on parkinsonism as an adverse drug event in order to evaluate the potential of a new predictive algorithm based on quantitative systems pharmacology (QSP). This is a ‘smart data’ approach, which, in contrast to ‘big data’ analytics that rely on high quality training sets, which are often not available or easily generalizable, is based on a computer model of relevant humanized neuronal circuits informed by formalized domain expertise and calibrated with clinical outcomes. The platform has shown value in predicting unexpected clinical efficacy and side-effect outcomes in schizophrenia (Geerts et al., 2012; Liu et al., 2014) and cognition in Alzheimer’s disease (Nicholas, 2013). In addition, the platform explicitly models the pharmacology of the two antipsychotics in a neurophysiological and neuropharmacological context at their correct level of target engagement and is well suited to quantitatively evaluate the non-linear pharmacodynamic interactions between the two drugs that affect the clinical outcome.

A frequent challenge in evaluating innovations of this type is the paucity of sets of sufficient quality from routine clinical practice to test such tools in predicting the risk for adverse drug reactions. Data derived from electronic health records (EHRs) offer potential solutions, since they include diverse and rich clinical information (Perera et al., 2016; Stewart et al., 2009), albeit requiring extraction from text fields. The objective of our study was to evaluate the accuracy with which the QSP platform, solely using information on antipsychotic names and doses in people receiving polypharmacy, could predict subsequently recorded parkinsonism.

## Methods

### Settings and data extraction

Data were extracted from the South London and Maudsley NHS Foundation Trust (SLAM) EHRs using the Clinical Record Interactive Search (CRIS) resource. SLAM is one of Europe’s largest mental healthcare providers, serving a catchment of four London boroughs (Lambeth, Southwark, Lewisham and Croydon) and a population of approximately 1.36 million (Perera et al., 2016; Stewart et al., 2009). The CRIS was developed in 2008 to allow researchers to search and retrieve anonymized SLAM EHRs and was approved as a data resource for secondary analysis by Oxford Research Ethics Committee C (reference 08/H606/71+5). At present, close to 400,000 cases are represented in the CRIS.

### Study sample

For the period between 1 January 2007 and 31 December 2014, we identified all patients who had received a diagnosis of schizophrenia (ICD-10 code: F20.x) who were in contact with SLAM clinical services, and who had been prescribed a combination of two antipsychotics.

### Cohort definition and characterization

The aim was to apply the predictive algorithms in a sample of people receiving APP. Antipsychotic medication data were extracted from several sources, including SLAM’s pharmacy-dispensing database, which mostly reflects medications prescribed on inpatient wards. In addition, we extracted data on recorded medication use from structured (e.g. drop-down menus) and free-text fields, such as correspondence and progress notes. Free-text was mined by a series of natural language processing (NLP) algorithms through General Architecture for Text Engineering software (Cunningham et al., 2013), which has been used to derive a large volume of meta-data in the CRIS for previous and current research (Kadra et al., 2015; Perera et al., 2016; Thompson et al., 2016). Data were extracted on all antipsychotic drugs listed in the British National Formulary (BNF) 65. For the purposes of this study, we ascertained patients who had been concurrently prescribed two antipsychotic medications for six or more months. The derivation of this exposure has been previously described in detail (Kadra et al., 2015); however, in brief, information about antipsychotic co-prescribing was extracted using a combination of NLP and a bespoke algorithm, with 94% positive predictive value (precision) and 60% sensitivity (recall). Information on antipsychotic dose was extracted from free-text using NLP, and from structured fields. APP cases where dose was not available for all antipsychotics that were part of the polypharmacy, and cases where the dose exceeded the maximum recommended dose in BNF prescribing guidelines, were not included.

### Outcome

We aimed to ascertain newly recorded parkinsonism after APP had been received for at least six months, having excluded anyone with a previous history of parkinsonism. With the help of senior clinicians, we compiled a parkinsonism dictionary including common alternative terms used clinically. Parkinsonism was extracted from free-text fields using the NLP pipeline, Adverse Drug Event annotation Pipeline ADEPt (Iqbal et al., 2017) with positive predictive value 89% and sensitivity 88%, respectively.

### Building a QSP-based classifier

The prediction of adverse drug events in this study was based on a neurophysiology-based computer model of human neuronal circuits, informed by formalized domain expertise that has been captured in mathematical equations (Roberts et al., 2016) (see Supplementary Material online). First, we simulate the competition of the two antipsychotics at their individual targets, mostly G-protein coupled receptors with their correct target engagement, modifying the activation levels of all the different central nervous systems (CNS) targets that are affected by the two drugs. The affinity for D_2_ and 5-HT_2A_R and data from positron emission tomography imaging studies are available in Supplementary Table 1 online and were derived from the standardized Psycho-active Drug Screening Protocol (Kroeze et al., 2015).

This QSP model is based on the known neuro-anatomical pathways linking supplemental motor area cortex to different parts of the dorsal striatum, including a striatal part with two types of medium spiny neurons (MSNs): D_1_+ MSN projecting into the globus pallidus interna of the direct pathway; D_2_+ MSNs projecting into the globus pallidus externa and the subthalamic nucleus (STN) of the indirect pathway. As shown experimentally in patients with Parkinson’s disease scheduled for deep brain stimulation, local field potentials measured in the STN strongly correlate with hypokinetic symptoms of bradykinesia and rigidity. The same readout in the computer model was previously found to correlate strongly with extrapyramidal symptom (EPS) liability both in patients with schizophrenia receiving single antipsychotics in 34 drug–dose combinations and in patients with Parkinson’s disease treated with 22 different therapeutic agents (Roberts et al., 2016).

### Implementation of other classifiers

We also tested the predictive value of three other simple features. Chlorpromazine equivalents (Leucht et al., 2015, 2016; Woods, 2003) are popularly used in the prescription of antipsychotic medication. Using conversion tables, we used the sum of this measure across the two antipsychotics as a predictor for EPS liability. Another parameter that is proportional to the EPS liability is a combination of the affinities for the D_2_R (Spiros et al., 2013); here we calculated the predictor as (1/K_1_ + 1/K_2_). Finally, in order to get a slightly more biological value and to include the effect of the specific doses, we calculated the sum of the D_2_R occupancies for the combination of the two antipsychotics using our receptor competition model (Spiros et al., 2010).

### Statistical analysis

STATA 13 was used to conduct all statistical analyses. Area under the receiver operating characteristic curve (AUROC) statistics were used to describe the prediction of recorded parkinsonism in patients prescribed two antipsychotics for six or more months, for all four models (QSP, D_2_R, 1/K sum, chlorpromazine equivalent dose). We further compared the performance of the indicator in predicting parkinsonism side-effects with chance.

## Results

**Figure 1.**
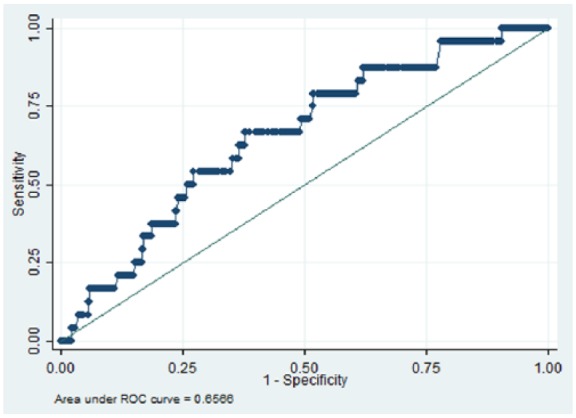
Area under the curve for predicting the risk for parkinsonism symptoms using Quantitative Systems Pharmacology. ROC: receiver operating characteristic.

**Figure 2.**
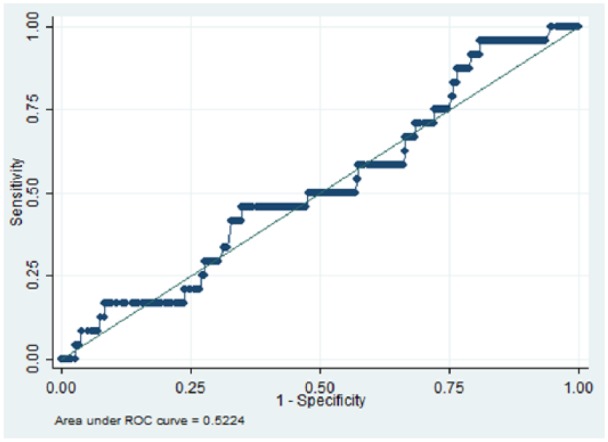
Area under the curve for predicting the risk for parkinsonism symptoms using D_2_R. ROC: receiver operating characteristic.

**Figure 3.**
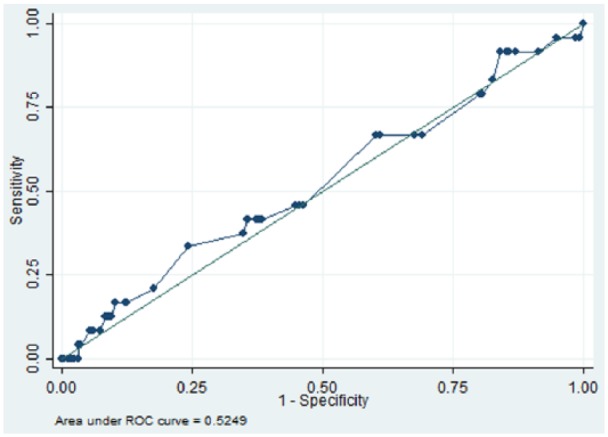
Area under the curve for predicting the risk for parkinsonism symptoms using 1/K_sum_. ROC: receiver operating characteristic

**Figure 4.**
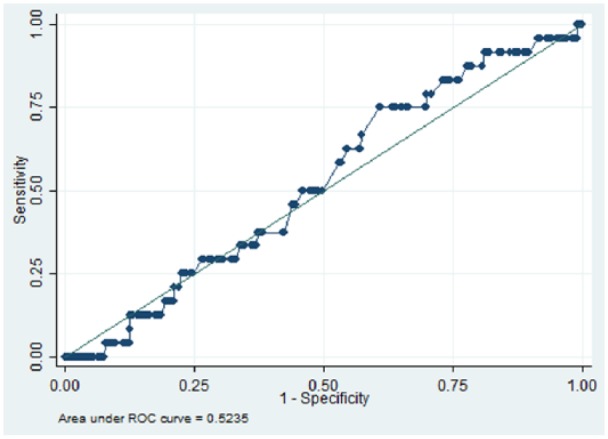
Area under the curve for predicting the risk for parkinsonism symptoms using chlorpromazine equivalence. ROC: receiver operating characteristic.

In total 832 patients with schizophrenia were ascertained who had been prescribed two antipsychotics for six or more months. Table 1 summarizes the demographic characteristics of the cohort. Overall, there was a higher proportion of patients aged between 26 and 35 (26.8), and 36 and 45 (26.9%) years. In relation to gender, a larger proportion of the group were male (61.5%), and more patients identified with British (32.9%) and Black African (32.2%) ethnicity. We identified 598 unique dose and antipsychotic combinations, and 59 unique antipsychotic combinations (not including dose). Table 2 summarizes the top five most frequent antipsychotic combinations. Overall, 24 (2.9%) patients were recorded as having parkinsonism after the defined period of receiving two antipsychotic medications. Figures 1–4 and Table 3 illustrate the AUROC output for all four models. In summary, QSP performed significantly better than chance in predicting parkinsonism (*p*=0.01), whereas predictions from D_2_R (*p*=0.350), 1/K_sum_ (*p*=0.347) and chlorpromazine equivalence (*p*= 0.330) were not statistically significant.

**Table 1.** Cohort demographic characteristics (*N* = 832).

Demographic characteristics	*n* (%)
**Age, years**	
16–25	207 (24.9)
26–35	223 (26.8)
36–45	224 (26.9)
46–55	106 (12.7)
56–65	47 (5.6)
66+	25 (3.0)
**Gender**	
Female	320 (38.5)
Male	512 (61.5)
**Ethnicity**	
British	274 (32.9)
Other White	62 (7.4)
Asian	56 (6.7)
Black Caribbean	92 (11.1)
Black African	268 (32.2)
Other	80 (9.6)

**Table 2.** Antipsychotic combinations.

Most common antipsychotic combinations^a^	*n* (%)
Aripiprazole and olanzapine	118 (14.11)
Olanzapine and risperidone	92 (11.00)
Amisulpride and clozapine	86 (10.3)
Amisulpride and sulpiride	57 (6.8)
Aripiprazole and clozapine	53 (6.3)
Other	430 (51.5)

a There were 59 different antipsychotic combinations (not including variation in dose).

**Table 3.** Receiver operating characteristic results of parkinsonism side-effects in patients prescribed two oral antipsychotics for six or more months (*N* = 832).

Index	Area under curve	Standard error	95% CI
**QSP**	0.66	0.05	0.55–0.76
**D_2_R**	0.52	0.06	0.41–0.64
**1/K_sum_**	0.53	0.06	0.40–0.65
**Chlorpromazine equivalence**	0.52	0.05	0.42–0.63

QSP: Quantitative Systems Pharmacology; CI: confidence interval

## Discussion

To our knowledge, this is the first example of a predictive classifier for APP outcomes in clinical practice using routine health records as a training set. The QSP classifier under evaluation was able to achieve reasonably good predictive value for parkinsonism in patients with schizophrenia who had been ascertained as receiving a combination of two antipsychotics for at least six months. In contrast, simpler approaches such as D_2_R, 1/K_sum_ and chlorpromazine equivalence were not able to achieve statistically significant prediction.

Previously, the QSP platform has been evaluated in schizophrenia and successfully predicted a clinical EPS side-effect for a new investigative drug that was not observed in preclinical animal models (Geerts et al., 2012) and an accurate effect size on total PANSS score for a drug that affected a completely novel target (Liu et al., 2014). Beyond the specific product under evaluation, the findings provide at least proof-of-concept evidence for the broader approach of utilizing neuronal circuit modelling to underpin clinical applications.

Our results reflect predictions based on minimal clinical information (antipsychotic names and doses) and we believe that adding further information such as age, gender and other medications prescribed in conjunction with the APP might further improve the predictive power of the model. For example, evidence indicates that benzodiazepines that increase GABA tone can also modulate EPS liability (Susatia and Fernandez, 2009), similar to serotoninergic modulation with antidepressants (Shireen, 2016). The QSP model has over 35 different CNS targets that encompass the pharmacology of most approved CNS active medications. Furthermore, the QSP model has also implemented the COMTVal156Met, APOE, 5-HTTLPR (rs23351) and D2DRTaq1A1 genotypes (Spiros & Geerts, 2012), thereby extending the nature of the ‘virtual’ patient.

This study had several important strengths. The QSP classifier is based on a formal implementation of domain expertise in neurophysiology, neuropharmacology, neuro-anatomy, neuro-imaging, calibrated with historical clinical data. Therefore, the model is able to provide an estimation of the expected clinical phenotype based on biological and pharmacological knowledge and in the absence of any training data. Here we tested the QSP model using a large and diverse cohort of people receiving routine secondary mental health services (Perera et al., 2016): a naturalistic, ‘real-world’ clinical cohort.

There are several potential limitations in this study, which should be borne in mind when drawing inferences. Data on parkinsonism and antipsychotic treatment relied on recorded information in the source health records. As previously highlighted in other work using the CRIS data resource (Iqbal et al., 2017; Kadra et al., 2015), we will not have captured all cases of parkinsonism because of below-100% sensitivity; therefore, it is likely that some predictions may have been underestimated. This study reports on motor side-effects only and did not seek to evaluate other clinical outcomes previously investigated, such as cognitive impairment (Geerts et al., 2015; Geerts et al., 2013) and clinical antipsychotic efficacy (Spiros et al., 2017). In this study we were unable to determine the reasons for co-prescribing two antipsychotics, therefore it is possible that in some cases, polypharmacy was co-prescribed due to failure to respond and/or adverse drug reactions.

Considering potential implications, advanced computer modelling approaches such as QSP might have future applicability as guides for clinicians in choosing the most appropriate treatment. This could conceivably be implemented as an interactive-interface clinical decision support such as alerts or reminders (Horsky et al., 2012), which could be integrated in the EHRs and can be flagged at the point clinicians enter new prescription information. Integrating this in EHR platforms would also mean that this system is available to all levels of secondary mental health care (e.g. outpatient and inpatient). More specifically, moving towards personalized medicine, the approach can be used to find the best-suited treatment strategy given patient characteristics, ultimately resulting in best effectiveness and lowest side-effects. Reducing adverse drug events is important in clinical practice as this might avoid adding anticholinergic medication, which in turn has been associated with detrimental outcomes such as decreased cognitive performance (Desmarais et al., 2014) and dementia (Risacher et al., 2016). We also believe that this modelling approach might facilitate clinical trial designs for novel drugs as it is a fundamental improvement over current pharmacokinetic/pharmacodynamic modelling approaches, which are based on small clinical datasets with relatively simple equations to predict the outcome in larger future trials. With a new target, there is no training or calibration set before the drug is actually tested in the clinical situation. The QSP model simulates the neurophysiology of the new target, thus could generate ‘virtual’ patients for drug development to test the impact of different genotypes and combinations of medications on dose–response.

## Supplemental Material

jop-2018-3453-File002 – Supplemental material for Predicting parkinsonism side-effects of antipsychotic polypharmacy prescribed in secondary mental healthcare Click here for additional data file. Supplemental material, jop-2018-3453-File002 for Predicting parkinsonism side-effects of antipsychotic polypharmacy prescribed in secondary mental healthcare by Giouliana Kadra, Athan Spiros, Hitesh Shetty, Ehtesham Iqbal, Richard D Hayes, Robert Stewart and Hugo Geerts in Journal of Psychopharmacology

## References

[bibr1-0269881118796809] Barnes TR (2011) Evidence-based guidelines for the pharmacological treatment of schizophrenia: Recommendations from the British Association for Psychopharmacology. J Psychopharmacol 25: 567–620. 21292923 10.1177/0269881110391123

[bibr2-0269881118796809] Citrome L (2013) A review of the pharmacology, efficacy and tolerability of recently approved and upcoming oral antipsychotics: An evidence-based medicine approach. CNS Drugs 27: 879–911. 24062193 10.1007/s40263-013-0105-7

[bibr3-0269881118796809] Cunningham H Tablan V Roberts A et al (2013) Getting more out of biomedical documents with GATE’s full lifecycle open source text analytics. PLoS Comput Biol 9: e1002854. 23408875 10.1371/journal.pcbi.1002854 PMC3567135

[bibr4-0269881118796809] Davis JM (1976) Comparative doses and costs of antipsychotic medication. Arch Gen Psychiatry 33: 858–861. 8024 10.1001/archpsyc.1976.01770070088010

[bibr5-0269881118796809] Desmarais JE Beauclair L Annable L et al (2014) Effects of discontinuing anticholinergic treatment on movement disorders, cognition and psychopathology in patients with schizophrenia. Ther Adv Psychopharmacol 4: 257–267. 25489477 10.1177/2045125314553611 PMC4257986

[bibr6-0269881118796809] Gallego JA Bonetti J Zhang J et al (2012) Prevalence and correlates of antipsychotic polypharmacy: A systematic review and meta-regression of global and regional trends from the 1970s to 2009. Schizophr Res 138: 18–28. 22534420 10.1016/j.schres.2012.03.018 PMC3382997

[bibr7-0269881118796809] Geerts H Roberts P Spiros A (2013) A quantitative system pharmacology computer model for cognitive deficits in schizophrenia. CPT Pharmacometrics Syst Pharmacol 2: e36. 23887686 10.1038/psp.2013.12 PMC3636495

[bibr8-0269881118796809] Geerts H Spiros A Roberts P (2015) Assessing the synergy between cholinomimetics and memantine as augmentation therapy in cognitive impairment in schizophrenia. A virtual human patient trial using quantitative systems pharmacology. Front Pharmacol 6: 198. 26441655 10.3389/fphar.2015.00198 PMC4585031

[bibr9-0269881118796809] Geerts H Spiros A Roberts P et al (2012) Blinded prospective evaluation of computer-based mechanistic schizophrenia disease model for predicting drug response. PLoS One 7: e49732. 23251349 10.1371/journal.pone.0049732 PMC3522663

[bibr10-0269881118796809] Grundy SM (2006) Drug therapy of the metabolic syndrome: Minimizing the emerging crisis in polypharmacy. Nat Rev Drug Discov 5: 295–309. 16582875 10.1038/nrd2005

[bibr11-0269881118796809] Horsky J Schiff GD Johnston D et al (2012) Interface design principles for usable decision support: A targeted review of best practices for clinical prescribing interventions. J Biomed Inform 45: 1202–1216. 22995208 10.1016/j.jbi.2012.09.002

[bibr12-0269881118796809] Iqbal E Mallah R Rhodes D et al (2017) ADEPt, a semantically-enriched pipeline for extracting adverse drug events from free-text electronic health records. PLoS One 12(11): e0187121. 29121053 10.1371/journal.pone.0187121 PMC5679515

[bibr13-0269881118796809] Joukamaa M Heliovaara M Knekt P et al (2006) Schizophrenia, neuroleptic medication and mortality. Br J Psychiatry 188: 122–127. 16449697 10.1192/bjp.188.2.122

[bibr14-0269881118796809] Kadra G Stewart R Shetty H et al (2015) Extracting antipsychotic polypharmacy data from electronic health records: Developing and evaluating a novel process. BMC Psychiatry 15: 166. 26198696 10.1186/s12888-015-0557-z PMC4511263

[bibr15-0269881118796809] Kadra G Stewart R Shetty H et al (2017) Antipsychotic polypharmacy prescribing and risk of hospital readmission. Psychopharmacology 235: 281–289. 29080904 10.1007/s00213-017-4767-6 PMC5748404

[bibr16-0269881118796809] Kennedy WK Jann MW Kutscher EC (2013) Clinically significant drug interactions with atypical antipsychotics. CNS Drugs 27: 1021–1048. 24170642 10.1007/s40263-013-0114-6

[bibr17-0269881118796809] Kroeze WK Sassano MF Huang XP et al (2015) PRESTO-Tango as an open-source resource for interrogation of the druggable human GPCRome. Nat Struct Mol Biol 22: 362–369. 25895059 10.1038/nsmb.3014 PMC4424118

[bibr18-0269881118796809] Kroken RA Johnsen E Ruud T et al (2009) Treatment of schizophrenia with antipsychotics in Norwegian emergency wards, a cross-sectional national study. BMC Psychiatry 9: 24. 19445700 10.1186/1471-244X-9-24 PMC2693495

[bibr19-0269881118796809] Leucht S Samara M Heres S et al (2015) Dose equivalents for second-generation antipsychotic drugs: The classical mean dose method. Schizophr Bull 41: 1397–1402. 25841041 10.1093/schbul/sbv037 PMC4601707

[bibr20-0269881118796809] Leucht S Samara M Heres S et al (2016) Dose equivalents for antipsychotic drugs: The DDD method. Schizophr Bull 42(Suppl. 1): S90–S94. 27460622 10.1093/schbul/sbv167 PMC4960429

[bibr21-0269881118796809] Liu J Ogden A Comery TA et al (2014) Prediction of efficacy of vabicaserin, a 5-HT2C agonist, for the treatment of schizophrenia using a quantitative systems pharmacology Model. CPT Pharmacometrics Syst Pharmacol 3: e111. 24759548 10.1038/psp.2014.7 PMC4011163

[bibr22-0269881118796809] Mace S Taylor D (2015) Reducing the rates of prescribing high-dose antipsychotics and polypharmacy on psychiatric inpatient and intensive care units: Results of a 6-year quality improvement programme. Ther Adv Psychopharmacol 5: 4–12. 25653825 10.1177/2045125314558054 PMC4315670

[bibr23-0269881118796809] Nicholas T Duvvuri S Leurent C et al (2013) Systems pharmacology modeling in neuroscience: Prediction and outcome of PF-04995274, a 5HT4 partial agonist, in a clinical scopolamine impairment trial. Adv Alzheimers Dis 2: 83–98.

[bibr24-0269881118796809] Perera G Broadbent M Callard F (2016) Cohort profile of the South London and Maudsley NHS Foundation Trust Biomedical Research Centre (SLaM BRC) case register: Current status and recent enhancement of an electronic mental health record-derived data resource. BMJ Open 6: e008721. 10.1136/bmjopen-2015-008721 PMC4785292 26932138

[bibr25-0269881118796809] Risacher SL McDonald BC Tallman EF et al (2016) Association between anticholinergic medication use and cognition, brain metabolism, and brain atrophy in cognitively normal older adults. JAMA Neurol 73: 721–732. 27088965 10.1001/jamaneurol.2016.0580 PMC5029278

[bibr26-0269881118796809] Roberts P Spiros A Geerts H (2016) A humanized clinically calibrated quantitative systems pharmacology model for hypokinetic motor symptoms in Parkinson’s Disease. Front Pharmacol 7: 6. 26869923 10.3389/fphar.2016.00006 PMC4735425

[bibr27-0269881118796809] Roh D Chang JG Kim CH et al (2013) Antipsychotic polypharmacy and high-dose prescription in schizophrenia: A 5-year comparison. Aust N Z J Psychiatry 48: 52–60. 23671214 10.1177/0004867413488221

[bibr28-0269881118796809] Shireen E (2016) Experimental treatment of antipsychotic-induced movement disorders. J Exp Pharmacol 8: 1–10. 27540314 10.2147/JEP.S63553 PMC4982503

[bibr29-0269881118796809] Spiros A Geerts H (2012) A quantitative way to estimate clinical off-target effects for human membrane brain targets in CNS Research and Development. J Exp Pharmacol 4: 53–62. 27186116 10.2147/JEP.S30808 PMC4863548

[bibr30-0269881118796809] Spiros A Carr R Geerts H (2010) Not all partial dopamine D(2) receptor agonists are the same in treating schizophrenia. Exploring the effects of bifeprunox and aripiprazole using a computer model of a primate striatal dopaminergic synapse. Neuropsychiatr Dis Treat 6: 589–603. 20856922 10.2147/NDT.S12460 PMC2938308

[bibr31-0269881118796809] Spiros A Roberts P Geerts H (2013) Phenotypic screening of the Prestwick library for treatment of Parkinson’s tremor symptoms using a humanized quantitative systems pharmacology platform. J Parkinsons Dis 3: 569–580. 24192755 10.3233/JPD-130211

[bibr32-0269881118796809] Spiros A Roberts P Geerts H (2017) Semi-mechanistic computer simulation of psychotic symptoms in schizophrenia with a model of a humanized cortico-striatal-thalamocortical loop. Eur Neuropsychopharmacol 27: 107–119. 28062203 10.1016/j.euroneuro.2016.12.006

[bibr33-0269881118796809] Stewart R Soremekun M Perera G et al (2009) The South London and Maudsley NHS Foundation Trust Biomedical Research Centre (SLAM BRC) case register: Development and descriptive data. BMC Psychiatry 9: 51. 19674459 10.1186/1471-244X-9-51 PMC2736946

[bibr34-0269881118796809] Susatia F Fernandez HH (2009) Drug-induced parkinsonism. Curr Treat Opt Neurol 11: 162–169. 10.1007/s11940-009-0019-3 19364450

[bibr35-0269881118796809] Thompson JV Clark JM Legge SE et al (2016) Antipsychotic polypharmacy and augmentation strategies prior to clozapine initiation: A historical cohort study of 310 adults with treatment-resistant schizophrenic disorders. J Psychopharmacol 30: 436–443. 26905920 10.1177/0269881116632376

[bibr36-0269881118796809] Torniainen M Mittendorfer-Rutz E Tanskanen A et al (2015) Antipsychotic treatment and mortality in schizophrenia. Schizophr Bull 41: 656–663. 25422511 10.1093/schbul/sbu164 PMC4393693

[bibr37-0269881118796809] Waddington JL Youssef HA Kinsella A (1998) Mortality in schizophrenia. Antipsychotic polypharmacy and absence of adjunctive anticholinergics over the course of a 10-year prospective study. Br J Psychiatry 173: 325–329. 9926037 10.1192/bjp.173.4.325

[bibr38-0269881118796809] Woods SW (2003) Chlorpromazine equivalent doses for the newer atypical antipsychotics. J Clin Psychiatry 64: 663–667. 12823080 10.4088/jcp.v64n0607

[bibr39-0269881118796809] Yu AP Ben-Hamadi R Birnbaum HG et al (2009) Comparing the treatment patterns of patients with schizophrenia treated with olanzapine and quetiapine in the Pennsylvania Medicaid population. Curr Med Res Opin 25: 755–764. 19199435 10.1185/03007990802683579

