# Supplementary material for: Predicting parkinsonism side-effects of antipsychotic polypharmacy prescribed in secondary mental healthcare
Source: J Psychopharmacol. 2018 Sep 20;32(11):1191–6. doi: 10.1177/0269881118796809 (PMC6238161; doi:10.1177/0269881118796809)
Supplement: jop-2018-3453-File002 – Supplemental material for Predicting parkinsonism side-effects of antipsychotic polypharmacy prescribed in secondary mental healthcare [file jop-2018-3453-File002.pdf]

Supplementary material

Because we have a limited number of patients with highly variable medications (at most 2 patients per unique combination), it is very challenging to generate a training or calibration. Our approach that does not need such a training set is based on a neurophysiology-based computer model of human neuronal circuits, informed by formalized domain expertise [1]. Basically this approach starts with simulating the competition of the two antipsychotics at their individual targets, mostly G-protein coupled receptors modifying the level of activation of all the different CNS targets that are affected by the two drugs [2]. The affinity for D_2 and 5-HT_{2A}R and data from PET imaging studies are listed in Table 1. In the absence of PET data for trifluoperazine and levomepromazine, we inferred equivalent doses based on their clinical comparison with known other first-generation antipsychotics. The affinities of antipsychotics and neurotransmitters for each human receptor subtype were derived from the standardized Psycho-active Drug Screening Protocol (PDSP) database (http://pdsp.med.unc.edu/indexR.html) [3].

Through their intracellular coupling with voltage-gated ion-channels, these receptor activation changes modulate the firing frequency of neuronal circuits in a biophysically realistic model of a closed cortico-striatal-thalamocortical model of the dorsal motor loop [1]. This QSP model is based on the known neuro-anatomical pathways linking Supplemental Motor Area-SMA cortex to different parts of the dorsal striatum, including a striatal part with two types of medium spiny neurons (MSN) : D_1+ MSN projecting into the Globus Pallidus Interna (GP_i) of the direct pathway, D_2+ MSN neurons projecting into the

Globus Pallidus Externa (GP_e) and the subthalamic Nucleus(STN) of the indirect pathway. The subthalamic nucleus projects back into the GP_i and the GP_i projects the combined activity out to the thalamus. A Thalamic Reticular Nucleus (TRN) subregion consists of GABAergic neurons and controls the activity projecting back into the motor cortex. There are 220 neurons of 8 different cell types with 21 GPCR targets implemented with their own particular coupling to voltage-gated ion channels. As shown experimentally [4] in Parkinson's patients scheduled for deep brain stimulation, the ratio of beta over gamma power of the local field potentials in the STN strongly correlates with hypokinetic symptoms of bradykinesia and rigidity. The calculated ratio of beta/gamma power in our computer model correlates strongly with both the EPS liability in schizophrenia patients with antipsychotics for 34 (single) drug-dose combinations and in Parkinson's patients treated with 22 different therapeutics [1].

Striatal dopaminergic hyperactivity associated with schizophrenia pathology was implemented quantitatively using results of a published PET imaging study that measures the basal dopamine level in human schizophrenia patients and controls [5] after transiently inhibiting dopamine synthesis with a-methyl-p-tyrosine. This leads to a a 6% increase in presynaptic DA release and a 65% increase in tonic firing frequency . Cortical schizophrenia pathology consists of reduced DA tone, NMDA and GABA dysfunction and increased noise, leading to a decrease in cognitive performance of 1.6 standard deviations below the normal population [6]. In addition, other

receptor densities changes as a consequence of chronic antipsychotic treatment have also been implemented ([7, 8]).

1. Roberts, P., A. Spiros, and H. Geerts, *A Humanized clinically calibrated quantitative systems pharmacology model for hypokinetic motor symptoms in Parkinson's Disease.* Frontiers in Pharmacology, 2016. **7**( ): p. 6.
2. Athan Spiros, H.G., *A quantitative way to estimate clinical off-target effects for human membrane brain targets in CNS Research and Development.* Journ. of Exp Pharmacology, 2012. **4**: p. 53-62.
3. Besnard, J., et al., *Automated design of ligands to polypharmacological profiles.* Nature, 2012. **492**(7428): p. 215-20.
4. Little, S., et al., *Adaptive deep brain stimulation in advanced Parkinson disease.* Ann Neurol, 2013. **74**(3): p. 449-57.
5. Abi-Dargham, A., et al., *Increased baseline occupancy of D2 receptors by dopamine in schizophrenia.* Proc Natl Acad Sci U S A, 2000. **97**(14): p. 8104-9.
6. Geerts, H., P. Roberts, and A. Spiros, *A quantitative system pharmacology computer model for cognitive deficits in schizophrenia.* CPT Pharmacometrics Syst Pharmacol, 2013. **2**: p. e36.
7. Geerts, H., et al., *Blinded prospective evaluation of computer-based mechanistic schizophrenia disease model for predicting drug response.* PLoS ONE, 2012. **7**(12): p. e49732.
8. Spiros, A., P. Roberts, and H. Geerts, *A Quantitative Systems Pharmacology Computer Model for Schizophrenia Efficacy and Extrapyramidal Side Effects.* Drug Development Research, 2012. **73:** (4): p. 196-213.
